# Protective Effects of *Pleurotus tuber-regium* on Carbon- Tetrachloride Induced Testicular Injury in Sprague Dawley Rats

**DOI:** 10.3389/fphar.2016.00480

**Published:** 2016-12-15

**Authors:** Kenneth O. Okolo, Iyeopu M. Siminialayi, Orish E. Orisakwe

**Affiliations:** ^1^Department of Pharmacology and Toxicology, Faculty of Pharmacy, Madonna UniversityPort Harcourt, Nigeria; ^2^Department of Pharmacology, Faculty of Basic Medical Sciences, College of Health Sciences, University of Port HarcourtPort Harcourt, Nigeria; ^3^Department of Experimental Pharmacology and Toxicology, Faculty of Pharmacy, University of Port HarcourtPort Harcourt, Nigeria

**Keywords:** male infertility, testicular toxicity, sex hormone, mushroom, carbon tetrachloride treatment

## Abstract

The high rate of male infertility and the meager resources to manage same in sub Saharan Africa have necessitated the search for cost effective and available alternatives. Mushrooms have been used traditionally in folk medicine and as nutraceuticals. This study has investigated the effect of the wild mushroom *Pleurotus tuber-regium* on carbon tetrachloride (CCl_4_) deleterious effects on the reproductive system of male rats. Thirty six rats were divided into six groups of six animals each. Group I (negative control) received 10 ml/kg olive oil intraperitoneal weekly in addition to feed and water *ad libitum*. Group II (positive control) received CCl_4_ 10 ml/kg (30% in Olive oil) weekly. Group III, IV, and V received 100 mg, 20 0mg, and 500 mg *P. tuber-regium* (33.3% in feed) daily in addition to 10 ml/kg CCl_4_ weekly. Group VI received 500 mg *P. tuber-regium* (33.3% in feed) daily. After 4 weeks, sperm motility, epididymal count and vitality were determined. Serum malondialdehyde (MDA), testosterone, Luteinizing hormone (LH), Follicle stimulating hormone (FSH), prolactin and oestradiol were estimated using enzyme-linked immunosorbent assay (ELISA) kits. Histopathologic examinations of the testis were carried out. Carbon tetrachloride significantly reduced the sperm motility (54.33 ± 3.79%), epididymal count (28.73 ± 2.86 × 10^6^/ml, vitality (4.96 ± 0.62), LH (0.88 ± 0.14), FSH (2.04 ± 0.33), and Testosterone (2.02 ± 0.24) when compared with control (89.33 ± 9.01), 91.91 ± 1.92 × 10^6^/ml, 13.12 ± 0.19, 2.74 ± 0.32, 3.64 ± 0.62, and 4.16 ± 0.23, respectively, which were reversed by *P. tuber-regium* administration. Co-administration of *P. tuber-regium* plus CCl_4_ significantly reduced MDA level. *P. tuber-regium* showed dose dependent ameliorative activity against CCl_4_ deleterious action on the testis and may be beneficial in the management of male infertility.

## Introduction

Infertility affects at least 20% of married couples and growing evidence suggest an increasing incidence of male reproductive problems (Kolettis, 2003; Nayernia et al., 2004). In Africa, infertility prevalence rates are feared to be higher and range from 20 to 35% (Eze and Okonofua, 2015). The “infertility belt,” geographical regions with high infertility prevalence, is well-known to Africa, stretching from West Africa, through Central to East Africa (Irvine, 1998; Larsen, 2000; Etuk, 2009). Sperm count declined to a mean of 71.2 million/ml in Ibadan, Nigeria, 54.6 million/ml in Lagos, Nigeria, 65.0 million/ml in Salem, Libya, 66.9 million/ml in Dar Es salaam and Tanzania (Elkin and Fenster, 1997). Nigeria has about twelve million infertile persons (Giwa-Osagie, 2003). Although there is a general documented belief that the most common cause of infertility in Nigeria is infection (Cates et al., 1985; Orisakwe et al., 2004), cases abound where infection have been treated without correction of infertility (Giwa-Osagie, 2003). Environmental pollution and indiscriminate use of herbal medications have tended to add burden on male infertility in sub Sahara Africa (Amadi et al., 2011; Orisakwe et al., 2014). In Nigeria there are higher rates of irreversible oligospermia or azoospermia than most other causes of infertility and less resources for the management of infertility (Osegbe and Amaku, 1985).

Mushrooms are regarded as functional foods and have been in use for decades in folk medicine. Most of the edible mushrooms belong to two major superfamily; Ascomcota and Basidiomycota (Dandapat and Sinha, 2015) with over 600 species possessing therapeutic activity (Wasser and Weis, 1999). Diabetic rats fed some mshroom fruiting bodies exhibit significant anti-glycemic and anti-hypercholesterolemic effects (Jeong et al., 2010; Volman et al., 2010). Similarly, mushrooms show positive influence on lipid metabolism, liver function and decreased severity of streptozotocin-induced diabetes in rats with considerable protective effects on the pancreas and apparent repopulation. Many edible mushrooms contain pharmacological active agents which are mainly secondary metabolites like alkaloids, tannins, polysaccharides, phenolics, flavonoids, etc. (Dandapat and Sinha, 2015). *Pleurotus tuber-regium* is an edible mushroom found in Nigeria (Okjuoya, 1991) with several medicinal and nutritional properties (Alobo, 2003; Hu et al., 2006; Ngai and Ng, 2006).

This study has evaluated the ameliorative effects of *P. tuber-regium* in carbon tetrachloride induced testicular damage in Sprague Dawley rats.

## Materials and Methods

### Harvesting of the Mushroom

Fresh fruiting bodies of wild *P. tuber-regium* were collected from a forest at the back of University of Nigeria Nsukka by a taxonomist working with the university. These fresh fruiting bodies were cleaned and air dried away from direct sunlight. The mushroom were ground and stored in a clean dry plastic container until use (El-kholy et al., 2013).

### Carbon Tetrachloride

Thirty percent carbon tetrachloride (Sigma–Aldrich) in Olive oil (Khan and Ahmed, 2009) was used to induce renal and hepatic damage at a dose of 10 ml/kg (i.p) (Karadeniz et al., 2009).

### Animal Husbandry

Thirty six male *Sprague-Dawley* rats with body weights 180–200 g acclimatized for 2 weeks were maintained under controlled conditions of temperature (23 ± 2°C) and humidity (50 ± 5%) and a 12-h light–dark cycle, were used for the experiment. The animals were housed in sanitized polypropylene cages containing sterile paddy husk as bedding. The bedding of the cages was changed daily and the cages were cleaned as well. They had free access to standard rat pellet diet and water *ad libitum*. The procedures were performed according to the guidelines on the use of animals and approved by the Institutional Animal Ethical Committee.

### Acute Toxicity Studies

Different concentrations of *P. tuber-regium* (50 – 5000 mg/kg b.w.) were administered orally to male rats. These animals were observed daily for toxicological manifestations like behavioral changes, neural and autonomic toxicities, feeding pattern changes, etc. There was no mortality recorded during this period even up to the dose of 5000 mg/kg (OECD, 1994).

### Experimental Design

The animals were divided into six groups with each group consisting of six animals each. The animals were treated as follows;

Group I – normal control received olive oil 10 ml/kg i.p. weekly in addition to standard food and water.
Group II – Positive control received CCl_4_ (30% CCl4 in olive oil) at a dose of 10 ml/kg weekly in addition to standard feed and water.
Group III – rats were treated orally with 100 mg/kg b.w. of *P. tuber-regium* in feed (33.3% w/w) along with 10 ml/kg CCl_4_ (30% in olive oil) weekly.
Group IV – rats were treated orally with 200 mg/kg b.w. of *P. tuber-regium* in feed (33.3% w/w) along with 10 ml/kg CCl_4_ (30% in olive oil) weekly.
Group V – rats were treated orally with 500 mg/kg b.w. of *P. tuber-regium* in feed (33.3% w/w) along with 10 ml/kg CCl_4_ (30% in olive oil) weekly.
Group VI – rats were treated orally with 500 mg/kg b.w. of *P. tuber-regium* in feed (33.3% w/w) only with standard feed and water.

### Necropsy

Treatments continued for 4 weeks. Blood was collected by retero orbital sinus puncture and serum was separated by centrifugation at 3000 r.p.m. Rats were sacrificed under ether anesthesia; the testis was excised, weighed, rinsed clean in saline, weighed and preserved in 10% formalin for histopathological study. Sperm was harvested from the caudal epididymis and mounted on a slide to determine sperm motility at 40× magnification. The motility assessment was expressed as percentage motile forms. The epididymal filtrate was then mixed in equal volume with eosin-nigrosin stain and a smear made of it was used for epididymal sperm vitality (Lasley et al., 1944). The caudal epididymal sperm reserve was determined using standard hemocytometric method (Amann and Almquist, 1961).

### Lipid Peroxidation Assay

Serum lipid peroxidation was quantified as malondialdehyde (MDA) according to the method described by Ohkawa et al. (1979). The MDA level was calculated according to the method of Todorova et al. (2005) and expressed as μmol MDA/mg protein.

### Sex Hormonal Assay

Serum levels of testosterone, Luteinizing hormone (LH), Follicle stimulating hormone (FSH), prolactin and oestradiol were estimated using enzyme-linked immunosorbent assay (ELISA) kits (Diagnostic System Laboratories Inc., USA), according to the manufacturer’s instruction.

### Statistical Analysis

Data were expressed as mean ± SD of the number of animals used in each group of the experiment. One way analysis of variance (ANOVA) was used to analyze the difference among the groups followed by Bonferroni’s posttest. Values of *p* < 0.05 were considered significant. Graph pad prism 5.0 software was used for statistical analysis.

## Results

**Table 1** shows the effect of *P. tuber-regium* on the testis and body weight CCl_4_ treated rats administration and treatment on the body weight of rats. Carbon tetrachloride significantly decreased the body weight [from 221 ± 2.24 (control) to 193 ± 1.87 g (CCl_4_ treated groups)] and increased the absolute (from 1.73 ± 0.40 (control) to 2.74 ± 0.12 g CCl4 group and relative weight of the testis [0.91 ± 0.20 (control) to 1.40 ± 0.02 CCl4 treated group *p* < 0.01]. Treatment with 100, 200, and 500 mg *P. tuber-regium* gave significant and dose-dependent reversal in the body weight and testis weight in CCl_4_ only treated group.

**Table 1:** Effect of *Pleurotus tuber-regium* on the testis and body weight CCl_4_ treated rats.

Groups	Mean body weight (g)				
			
	Initial	Final	Percent body weight change	Absolute weight of testis (g)	Relative weight testis
CONTROL	195 ± 1.87	221 ± 2.24	1.133	1.73 ± 0.40	0.91 ± 0.20
CCL_4_ Only	194 ± 2.30	193 ± 1.87^∗∗∗^	-0.005	2.74 ± 0.12^∗∗^	1.40 ± 0.02^∗∗^
CCL_4_ + 100 mg P.t.	193 ± 1.58	198 ± 1.58^∗∗∗^^##^	1.026	2.21 ± 0.03	1.15 ± 0.02
CCL_4_ + 200 mg P.t.	194 ± 2.50	202 ± 2.12^∗∗∗^^###^	1.041	2.31 ± 0.21	1.20 ± 0.11
CCL_4_ + 500 mg P.t.	196 ± 2.77	218 ± 0.89^###^	1.112	1.84 ± 0.40^##^	0.96 ± 0.25^##^
500 mg P.t. Only	194 ± 2.20	222 ± 1.86^###^	1.144	1.85 ± 0.20^##^	0.91 ± 0.20^##^


*Each value represents mean ± SD, *n* = 6. Values marked with an asterisk (^∗^) differ significantly from control value (^∗∗^*p* < 0.01, ^∗∗∗^*p* < 0.001) while those marked with # differ significantly from CCl_*4*_ only group (^##^*p* < 0.01, ^###^*p* < 0.001).*

The effect of *P. tuber-regium* on sperm motility, epididymal count, vitality and (MDA of CCl_4_ treated rats is shown on **Table 2**. Carbon tetrachloride significantly reduced the sperm motility (54.33 ± 3.79%), epididymal count (28.73 ± 2.86 × 10^6^/ml and vitality (4.96 ± 0.62) when compared with control (89.33 ± 9.01), 91.91 ± 1.92 × 10^6^/ml and 13.12 ± 0.19, respectively. There was significant increase in the sperm motility, epididymal count and vitality following *P. tuber-regium* administration. The reversal effect of *P. tuber-regium* on these seminal parameters were dose-dependent. There was significant difference between the MDA level (14.00 ± 3.50) in the CCl_4_ treated rats and the untreated control (1.40 ± 0.32 μg/mg protein). Following the co-administration of 100, 200, and 500 mg *P. tuber-regium* plus CCl_4_ there was significant reduction in the level of the MDA (3.50 ± 0.61, 1.60 ± 0.36, and 1.70 ± 0.15 μmol/mg protein, respectively) in dose-dependent manner.

**Table 2:** Effect of *P. tuber-regium* on sperm motility, epididymal count, vitality and malondialdehyde (MDA) of CCl_4_ treated rats.

GROUPS	Sperm Motility (%)	Caudal epididymal sperm count (10^6^/ml)	Sperm vitality (Live:Death ratio)	MDA (μmol/mg)
Control	89.33 ± 9.01	91.91 ± 1.92	13.12 ± 0.19	1.40 ± 0.32
CCl_4_ Only	54.33 ± 3.79^∗∗∗^	28.73 ± 2.86^∗∗∗^	4.96 ± 0.62^∗∗∗^	14.00 ± 3.50^∗∗∗^
CCl_4_ + 100 mg P.t.	72.33 ± 6.43	44.17 ± 3.23^∗∗∗#^	7.54 ± 0.14^∗∗∗###^	3.50 ± 0.61^###^
CCl_4_ + 200 mg P.t.	79.33 ± 8.08^#^	65.29 ± 4.302^∗∗∗###^	10.68 ± 0.18^∗∗∗^^###^	1.60 ± 0.36^∗∗∗^
CCl_4_ + 500 mg P.t.	79.33 ± 3.22^#^	78.30 ± 8.62^∗###^	11.21 ± 0.20^∗∗∗###^	1.70 ± 0.15^###^
500 mg P.t.	90.00 ± 8.68^###^	91.37 ± 1.64^###^	13.74 ± 0.58^###^	–


*Each value represents mean ± SD, *n* = 6. Values marked with an asterisk (^∗^) differ significantly from control value (^∗^*p* < 0.05, ^∗∗∗^*p* < 0.001) while those marked with # differ significantly from CCl_*4*_ only group (^#^*p* < 0.05, ^###^*p* < 0.001).*

**Table 3** shows the effect of *P. tuber-regium* on LH, FSH, Testosterone, Prolactin, and Estradiol of CCl_4_ treated rats. Administration of carbon tetrachloride significantly decreased the levels of LH (0.88 ± 0.14), FSH (2.04 ± 0.33), and Testosterone (2.02 ± 0.24) compared to control 2.74 ± 0.32, 3.64 ± 0.62, and 4.16 ± 0.23, respectively. The CCl_4_, however, increased significantly the levels of prolactin from 1.06 ± 0.21 to 3.58 ± 0.48 and oestradiol from 0.81 ± 0.10 (control) to 2.89 ± 0.18 in the CCl_4_ treated group. There was dose-dependent reversal of the effects of CCl4 on these hormonal parameters in the *P. tuber-regium* treated groups.

**Table 3:** Effect of *P. tuber-regium* on FSH, LH, Testosterone, Prolactin, and Estradiol of CCl_4_ treated rats.

GROUPS	Luteinizing hormone (LH) (mIU/ml)	Follicle stimulating hormones (FSHs) (mIU/ml)	Testosterone (ng/ml)	Prolactin (ng/ml)	Estradiol (pg/ml)
Control	2.74 ± 0.32	3.64 ± 0.62	4.16 ± 0.23	1.06 ± 0.21	0.81 ± 0.10
CCl_4_ Only	0.88 ± 0.14^∗∗∗^	2.04 ± 0.33^∗∗∗^	2.02 ± 0.24^∗∗∗^	3.58 ± 0.48^∗∗∗^	2.89 ± 0.18^∗∗∗^
CCl_4_ + 100 mg P.t.	1.31 ± 0.15^∗∗∗^	2.73 ± 0.17^∗^	2.30 ± 0.22^∗∗∗^	3.01 ± 0.20^∗∗∗^	2.69 ± 0.12^∗∗∗^
CCl_4_ + 200 mg P.t.	1.80 ± 0.16^∗∗∗###^	3.08 ± 0.13^##^	2.88 ± 0.26^∗∗∗###^	2.66 ± 0.27^∗∗∗##^	1.84 ± 0.23^∗∗∗###^
CCl_4_ + 500 mg P.t.	2.10 ± 0.16^∗∗###^	3.28 ± 0.30^##^	3.42 ± 0.22^∗∗###^	1.95 ± 0.41^∗∗###^	1.08 ± 0.10^###^
500 mg P.t.	2.73 ± 0.31^###^	3.50 ± 0.64^###^	4.28 ± 0.48^###^	1.14 ± 0.18^###^	0.83 ± 0.12^###^


*Each value represents mean ± SD, *n* = 6. Values marked with an asterisk (^∗^) differ significantly from control value (^∗^*p* < 0.05, ^∗∗^*p* < 0.01, ^∗∗∗^*p* < 0.001) while those marked with (#) differ significantly from CCl_*4*_ only group (^##^*p* < 0.01, ^###^*p* < 0.001).*

### Effect of *P. tuber-regium* on Histology of the Testis

**FIGURE 1:**
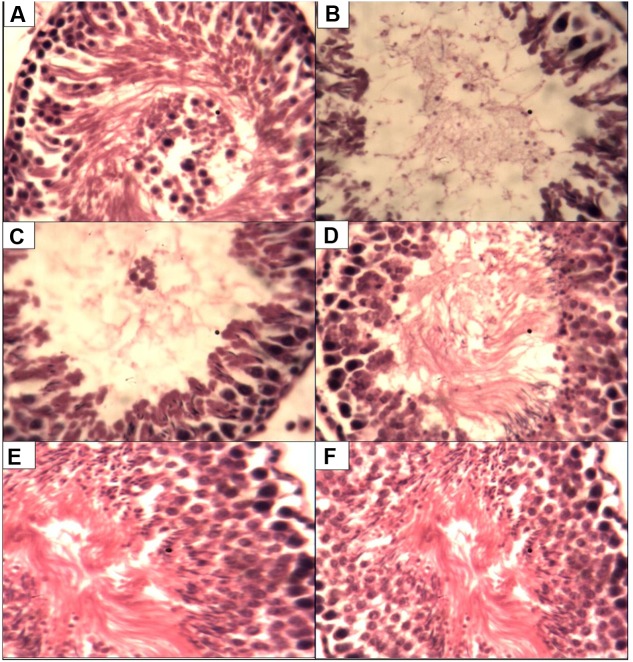
**Histopathological changes in the testes of rats (magnification H & E ×400).** **(A)** Control testis showing normal histology. **(B)** CCU only group showing with scanty spermatogenic cells. **(C)** CCU + l00 mg P.t. group showing seminiferous tubules without full population of spermatozoa. **(D)** CCU + 200 mg P.t. group showing seminiferous tubules without full population. **(E)** Received CCl4 + Pleurotus tuber-regium at a dose of 500 mg b.w. daily. **(F)** Received 500 mg Pleurotus tuber-regium only, i.e., treated negative control.

**Figure 1** present the histological changes in the CCl_4_ exposed animals. Administration of CCl_4_ to the animals lead to degeneration of the seminiferous tubules with different degrees of sperm cell arrest when compared to control. However, treatments with *P. tuber-regium* lead to improvement in sperm cell population while the 500 mg *P. tuber-regium* only group had there seminiferous tubules and sperm cell population similar to control.

## Discussion

Infertility which is usually defined as a couple’s inability to achieve pregnancy following 1 year of unprotected intercourse and considered one of the main public health issues (Jungwirth et al., 2012). Male factor infertility is present in approximately half of all infertile couples (Thonneau et al., 1991). Spermatogenesis takes place in the seminiferous tubular structures of the testis which are lined with germ and sertoli cells. Spermatogenesis consists of three major phases; Proliferation, reduction-division and differentiation with distinct and specific germ cell types being associated with each phase (Hess, 1990). The present study has investigated the effect of *P. tuber-regium* on carbon tetrachloride induced testicular injury in animal model. After hepatic handling CCl_4_ generates chloride and trichloromethyl radicals CCl_3_, which reacts with oxygen to produce CCl_3_ O_2_. CCl_3_ bind to fatty acids to generate alkoxy and peroxy radicals (Bruckner et al., 2002). The increased lipid peroxidation provoke destruction of sperm (Aitken et al., 2014). *P. tuber-regium* co-administered with carbon tetrachloride produced a significant dose dependent decrease in the level of MDA. Reduced lipid peroxidation will promote spermatogenesis.

There are estrogen receptors in the hypothalamic nuclei and in pituitary gonadotropes, which act on the hypothalamus to affect gonadotrophin releasing hormone GnRH pulses and at the pituitary level to regulate FSH and LH secretion (Cabler et al., 2010). The excessive conversion of testosterone to estrogen in peripheral adipose tissue has been known to cause secondary hypogonadism through hypothalamic-pituitary-gonadal axis inhibition (Cabler et al., 2010). Estrogens affect spermatogenesis directly within the testis by alterations in gonadotropin secretion by the pituitary gland. High levels of circulating oestradiol and/or elevated oestradiol/testosterone ratios which is a feature of male infertility (Cabler et al., 2010), was observed in rats treated with CCl_4_ in this study. *P. tuber-regium* significantly reversed the oestradiol level in this study. Although there is a school of thought that normal levels of gonadotropins in the context of low free testosterone may signify the effective suppression of the hypothalamic-pituitary axis, resulting in subclinical hypogonadotropic hypogonadism (Agarwal et al., 2006). In this study, however, *P. tuber-regium* significantly reversed the CCl_4_, LH, FSH, and testosterone lowering and oestradiol increase. *P. tuber-regium* may be competitively bind to estrogen receptors on the hypothalamus and pituitary gland to decrease estrogenic firing to increase the release of LH, which increases testosterone production by the testes.

Luteinizing hormone binds to its receptors to activate G-proteins and, in turn, adenylate cyclase, which can increase cyclic AMP formation. cAMP will then stimulate protein kinase A (PKA), which will phosphorylate proteins. The phosphorylated proteins will further phosphorylate other proteins or induce new protein synthesis, i.e., steroidogenic acute regulatory protein (Manna et al., 2013). The function of steroidogenic acute regulatory protein is to transfer free cholesterol from the cytoplasm into the inner membrane of mitochondria, where cytochrome P450 side-chain cleavage enzyme converts cholesterol to pregnenolone (Stocco, 2002; Manna and Stocco, 2005). Pregnenolone will then be transported to smooth endoplasmic reticulum for testosterone synthesis, which is an essential steroid hormone for reproduction in males (Liu et al., 2007). It has also been shown that activation of the protein kinase C (PKC) signal pathway can strongly modulate Leydig cell steroidogenesis (Hirakawa et al., 2002). Chen et al. (2005) explained that *Clonorchis sinensis* activated both PKA and PKC signal transduction pathways to stimulate cell steroidogenesis. Although there seem to be no significant association between semen parameters and prolactin levels (Lotti et al., 2013), prolactin can affect steroidogenesis by modulating the expression of LH receptors (Dombrowicz et al., 1992), or by regulating the activity of steroidogenetic enzymes (Chandrashekar and Bartke, 1988) and has a trophic effect on male seminal accessory glands.

Histological examination suggests the consequences of oxidative stress in spermatogenesis with sperm cell maturation arrest. The result conform with the sperm parameter result of decreased sperm count since maturation arrest will ultimately decrease the number of sperm reaching the epididymis (El-kholy et al., 2013).

## Conclusion

In this study has shown that *P. tuber-regium* may protect the testis from CCl_4_ induced damage as evidenced by the by the ameliorative effects on the epididymal sperm count, motility, viability, sex hormones, MDA, and testicular histomorphology. Since biochemical hypogonadism is considered a main feature of male infertility, dietary supplementation with *P. tuber-regium* with its significant reversal of all the deleterious testicular effects in CCl_4_ treated rats may hold some promise in the management of involuntary childlessness amongst married couples.

## Author Contributions

KO: Designed study, carried out the bench work and analysed data, IS: Design, and OO: Designed the study, analysed data and write up.

## Conflict of Interest Statement

The authors declare that the research was conducted in the absence of any commercial or financial relationships that could be construed as a potential conflict of interest.
